# *N*′-[(*E*)-4-Bromo­benzyl­idene]pyrazine-2-carbohydrazide

**DOI:** 10.1107/S1600536813016917

**Published:** 2013-06-22

**Authors:** Shahid Hameed, Mushtaq Ahmad, M. Nawaz Tahir, Muhammad Abdullah Shah, Hazoor Ahmad Shad

**Affiliations:** a Department of Chemistry, Quaid-i-Azam University, Islamabad, Pakistan; b Medicinal Botanic Centre, PCSIR Laboratories Complex, Peshawar, Pakistan; c Department of Physics, University of Sargodha, Sargodha, Pakistan; d Department of Chemistry, University of Hazara, Mansehra, Pakistan; e Department of Chemistry, University of Sargodha, Sargodha, Pakistan

## Abstract

In the title compound, C_12_H_9_BrN_4_O, the *N*′-methyl­idene­pyrazine-2-carbohydrazide and 4-bromobenzene groups are oriented at a dihedral angle of 10.57 (7)°. The hydrazide N—H group is involved in intra­molecular N—H⋯N inter­action, which generates an *S*(5) motif. A short C—H⋯O inter­action is formed between the methyl­idene H atom and the carbonyl O atom. It connects mol­ecules into chains extending along [100]. In addition, mol­ecules are arranged into stacks extending along [010] *via* π–π inter­actions between pyrazine and benzene rings, with centroid–centroid distances of 3.837 (2) and 3.860 (2) Å.

## Related literature

For a related crystal structure and related studies, see: Hearn & Cynamon (2004 ▶); Jin *et al.* (2006 ▶); Yuan *et al.* (2006 ▶). For graph-set notation, see: Bernstein *et al.* (1995 ▶).
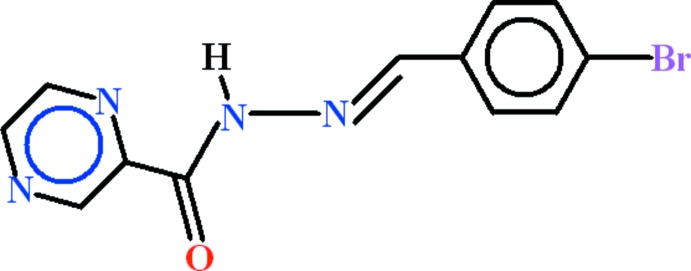


## Experimental

### 

#### Crystal data

C_12_H_9_BrN_4_O
*M* *_r_* = 305.14
Triclinic, 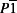

*a* = 5.8947 (9) Å
*b* = 7.6941 (12) Å
*c* = 14.029 (2) Å
α = 83.273 (7)°
β = 80.086 (7)°
γ = 72.440 (6)°
*V* = 596.11 (16) Å^3^
*Z* = 2
Mo *K*α radiation
μ = 3.44 mm^−1^
*T* = 296 K
0.26 × 0.22 × 0.20 mm

#### Data collection

Bruker Kappa APEXII CCD diffractometer
Absorption correction: multi-scan (*SADABS*; Bruker, 2005 ▶) *T*_min_ = 0.425, *T*_max_ = 0.503
7382 measured reflections
2529 independent reflections
1403 reflections with *I* > 2σ(*I*)
*R*_int_ = 0.074

#### Refinement

*R*[*F* ^2^ > 2σ(*F* ^2^)] = 0.039
*wR*(*F* ^2^) = 0.084
*S* = 0.94
2529 reflections
163 parameters
H-atom parameters constrained
Δρ_max_ = 0.28 e Å^−3^
Δρ_min_ = −0.30 e Å^−3^

### 

Data collection: *APEX2* (Bruker, 2007 ▶); cell refinement: *SAINT* (Bruker, 2007 ▶); data reduction: *SAINT*; program(s) used to solve structure: *SHELXS97* (Sheldrick, 2008 ▶); program(s) used to refine structure: *SHELXL97* (Sheldrick, 2008 ▶); molecular graphics: *ORTEP-3 for Windows* (Farrugia, 2012 ▶) and *PLATON* (Spek, 2009 ▶); software used to prepare material for publication: *WinGX* (Farrugia, 2012 ▶) and *PLATON*.

## Supplementary Material

Crystal structure: contains datablock(s) global, I. DOI: 10.1107/S1600536813016917/gk2580sup1.cif

Structure factors: contains datablock(s) I. DOI: 10.1107/S1600536813016917/gk2580Isup2.hkl

Supplementary material file. DOI: 10.1107/S1600536813016917/gk2580Isup3.cml

Additional supplementary materials:  crystallographic information; 3D view; checkCIF report

## Figures and Tables

**Table 1:** Hydrogen-bond geometry (Å, °)

*D*—H⋯*A*	*D*—H	H⋯*A*	*D*⋯*A*	*D*—H⋯*A*
C6—H6⋯O1^i^	0.93	2.24	3.132 (4)	161
N3—H3*A*⋯N1	0.86	2.27	2.671 (3)	108

Symmetry code: (i) 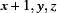.

**Fig. 1.**
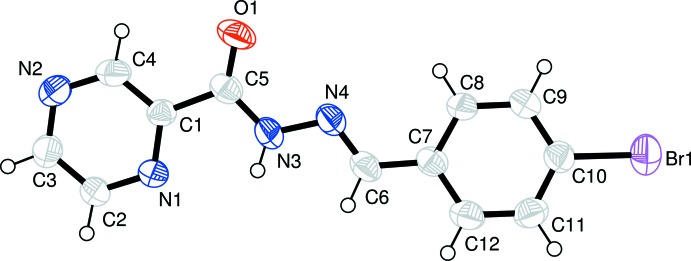
View of the title compound with the displacement ellipsoids drawn at the 50% probability level. H atoms are shown by small circles of arbitrary radii.

**Fig. 2.**
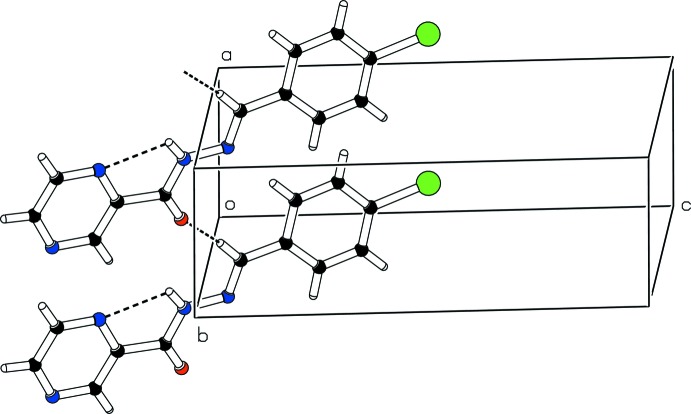
Packing diagram of the title compound showing S(5) motif and C(6) chains along [1 0 0].

## supplementary crystallographic information

### Comment

The title compound (Fig. 1) was prepared to study biological activities of hydrazone compounds (Hearn & Cynamon, 2004; Jin *et al.*, 2006).

Crystals of the earlier reported 4-chlorobenzaldehyde(pyrazine-2-carbonyl) hydrazone (Yuan *et al.*, 2006) are practically isostructural with the title compound.

In the title compound the N'-methylidenepyrazine-2-carbohydrazide (A) (C1–C6/N1–N4/O1) and 4-bromophenyl (B) (C7–C12/Br1) moieties are almost planar with r. m. s. deviations of 0.061 Å and 0.009 Å, respectively. The dihedral angle between A/B is 10.57 (7)°. There exists intramolecular N—H···.N hydrogen bond (Table 1, Fig. 2) forming *S*(5) motif (Bernstein *et al.*, 1995). The intermolecular hydrogen bonds of C—H···.O type (Table 1, Fig. 2) generate *C*(6) chains (Bernstein *et al.*, 1995) along the crystallographic *a*-axis. There exist π–π interactions with a distance of 3.838 (2) Å [*Cg*1—*Cg*2^i^ & *Cg*2— *Cg*1^i^: i = -*x*, 2 - *y*, -*z*] and 3.860 (2) Å [*Cg*1—*Cg*2^ii^ & *Cg*2—*Cg*1^ii^: ii = -*x*, 1 - *y*, -*z*], between the centroids of pyrazine (*Cg*1) and benzene (*Cg*2) rings.

### Experimental

The title compound was prepared by the condensation of equimolar ratio of pyrazine-2-carbohydrazide (0.50 g, 3.6 mmol) and 4-bromobenzaldehyde (0.67 g, 3.6 mmol) in methanol by the reflux of 5 h. The resulting reaction mixture was allowed to cool over night. The precipitated solid was filtered, washed with petroleum ether and recrystalized from chloroform in petroleum ether and dried under reduced pressure over CaCl_2_ to give white prisms. Rf: 0.40 (30% acetone in petroleum ether): Yield: 83%, soluble in chloroform; m.p. 546–547 K.

### Refinement

The H atoms were positioned geometrically (N–H = 0.86 Å, C–H = 0.93 Å) and refined as riding on their carriers with *U*_iso_(H) = *xU*_eq_(C, N), where *x* = 1.2 for all H-atoms.

### Crystal data

**Table:**

C_12_H_9_BrN_4_O	*Z* = 2
*M**_r_* = 305.14	*F*(000) = 304
Triclinic, *P*1	*D*_x_ = 1.700 Mg m^−^^3^
Hall symbol: -P 1	Mo *K*α radiation, λ = 0.71073 Å
*a* = 5.8947 (9) Å	Cell parameters from 1403 reflections
*b* = 7.6941 (12) Å	θ = 2.1–25.5°
*c* = 14.029 (2) Å	µ = 3.44 mm^−^^1^
α = 83.273 (7)°	*T* = 296 K
β = 80.086 (7)°	Prism, white
γ = 72.440 (6)°	0.26 × 0.22 × 0.20 mm
*V* = 596.11 (16) Å^3^	

### Data collection

**Table:**

Bruker Kappa APEXII CCD diffractometer	2529 independent reflections
Radiation source: fine-focus sealed tube	1403 reflections with *I* > 2σ(*I*)
Graphite monochromator	*R*_int_ = 0.074
Detector resolution: 8.10 pixels mm^-1^	θ_max_ = 27.1°, θ_min_ = 1.5°
ω scans	*h* = −7→6
Absorption correction: multi-scan (*SADABS*; Bruker, 2005)	*k* = −9→9
*T*_min_ = 0.425, *T*_max_ = 0.503	*l* = −17→17
7382 measured reflections	

### Refinement

**Table:**

Refinement on *F*^2^	Primary atom site location: structure-invariant direct methods
Least-squares matrix: full	Secondary atom site location: difference Fourier map
*R*[*F*^2^ > 2σ(*F*^2^)] = 0.039	Hydrogen site location: inferred from neighbouring sites
*wR*(*F*^2^) = 0.084	H-atom parameters constrained
*S* = 0.94	*w* = 1/[σ^2^(*F*_o_^2^) + (0.0355*P*)^2^] where *P* = (*F*_o_^2^ + 2*F*_c_^2^)/3
2529 reflections	(Δ/σ)_max_ < 0.001
163 parameters	Δρ_max_ = 0.28 e Å^−^^3^
0 restraints	Δρ_min_ = −0.30 e Å^−^^3^

### Special details

**Table:**

Geometry. All e.s.d.'s (except the e.s.d. in the dihedral angle between two l.s. planes) are estimated using the full covariance matrix. The cell e.s.d.'s are taken into account individually in the estimation of e.s.d.'s in distances, angles and torsion angles; correlations between e.s.d.'s in cell parameters are only used when they are defined by crystal symmetry. An approximate (isotropic) treatment of cell e.s.d.'s is used for estimating e.s.d.'s involving l.s. planes.
Refinement. Refinement of *F*^2^ against ALL reflections. The weighted *R*-factor *wR* and goodness of fit *S* are based on *F*^2^, conventional *R*-factors *R* are based on *F*, with *F* set to zero for negative *F*^2^. The threshold expression of *F*^2^ > σ(*F*^2^) is used only for calculating *R*-factors(gt) *etc*. and is not relevant to the choice of reflections for refinement. *R*-factors based on *F*^2^ are statistically about twice as large as those based on *F*, and *R*- factors based on ALL data will be even larger.

### Fractional atomic coordinates and isotropic or equivalent isotropic displacement parameters (Å^2^)

**Table:**

	*x*	*y*	*z*	*U*_iso_*/*U*_eq_	
Br1	0.36405 (7)	0.25644 (5)	0.47485 (3)	0.07332 (19)	
O1	−0.4594 (4)	0.8146 (3)	−0.03918 (16)	0.0633 (7)	
N1	−0.0274 (4)	0.9547 (3)	−0.21291 (18)	0.0486 (7)	
N2	−0.4307 (5)	1.1093 (4)	−0.3071 (2)	0.0599 (8)	
N3	−0.0701 (5)	0.7949 (3)	−0.03380 (18)	0.0523 (7)	
H3A	0.0565	0.8198	−0.0649	0.063*	
N4	−0.0613 (5)	0.7038 (3)	0.05682 (19)	0.0508 (7)	
C1	−0.2458 (5)	0.9456 (4)	−0.1727 (2)	0.0401 (8)	
C2	−0.0148 (6)	1.0405 (4)	−0.3008 (2)	0.0538 (9)	
H2	0.1337	1.0501	−0.3321	0.065*	
C3	−0.2126 (6)	1.1151 (4)	−0.3467 (2)	0.0549 (9)	
H3	−0.1927	1.1724	−0.4084	0.066*	
C4	−0.4426 (6)	1.0230 (4)	−0.2191 (3)	0.0540 (9)	
H4	−0.5918	1.0148	−0.1877	0.065*	
C5	−0.2732 (6)	0.8455 (4)	−0.0745 (2)	0.0476 (8)	
C6	0.1460 (6)	0.6511 (4)	0.0827 (2)	0.0496 (9)	
H6	0.2748	0.6734	0.0398	0.060*	
C7	0.1898 (6)	0.5575 (4)	0.1764 (2)	0.0449 (8)	
C8	0.0083 (6)	0.5360 (4)	0.2494 (2)	0.0496 (8)	
H8	−0.1509	0.5833	0.2386	0.060*	
C9	0.0571 (6)	0.4466 (4)	0.3372 (2)	0.0524 (9)	
H9	−0.0679	0.4339	0.3853	0.063*	
C10	0.2919 (6)	0.3757 (4)	0.3537 (2)	0.0500 (8)	
C11	0.4775 (6)	0.3941 (4)	0.2827 (2)	0.0555 (9)	
H11	0.6361	0.3455	0.2939	0.067*	
C12	0.4264 (6)	0.4854 (4)	0.1945 (2)	0.0543 (9)	
H12	0.5516	0.4989	0.1467	0.065*	

### Atomic displacement parameters (Å^2^)

**Table:**

	*U*^11^	*U*^22^	*U*^33^	*U*^12^	*U*^13^	*U*^23^
Br1	0.0815 (3)	0.0777 (3)	0.0601 (3)	−0.0224 (2)	−0.0225 (2)	0.01461 (19)
O1	0.0580 (15)	0.0762 (17)	0.0622 (15)	−0.0398 (13)	0.0071 (13)	0.0006 (12)
N1	0.0420 (16)	0.0576 (17)	0.0482 (17)	−0.0216 (13)	−0.0063 (13)	0.0069 (13)
N2	0.0479 (18)	0.070 (2)	0.064 (2)	−0.0208 (15)	−0.0186 (16)	0.0107 (15)
N3	0.0564 (19)	0.0604 (18)	0.0429 (16)	−0.0271 (14)	−0.0057 (15)	0.0106 (13)
N4	0.0555 (19)	0.0552 (17)	0.0433 (17)	−0.0238 (14)	−0.0035 (14)	0.0059 (13)
C1	0.0373 (19)	0.0392 (18)	0.0455 (19)	−0.0143 (15)	−0.0058 (16)	−0.0009 (14)
C2	0.048 (2)	0.066 (2)	0.050 (2)	−0.0259 (17)	−0.0076 (18)	0.0125 (17)
C3	0.057 (2)	0.061 (2)	0.051 (2)	−0.0225 (18)	−0.0159 (19)	0.0060 (17)
C4	0.041 (2)	0.062 (2)	0.062 (2)	−0.0221 (17)	−0.0034 (18)	−0.0014 (18)
C5	0.052 (2)	0.047 (2)	0.048 (2)	−0.0234 (17)	−0.0001 (18)	−0.0048 (16)
C6	0.055 (2)	0.053 (2)	0.045 (2)	−0.0293 (17)	−0.0008 (18)	0.0027 (16)
C7	0.048 (2)	0.0429 (18)	0.048 (2)	−0.0225 (15)	−0.0039 (17)	−0.0031 (15)
C8	0.0401 (19)	0.053 (2)	0.057 (2)	−0.0192 (16)	−0.0062 (17)	0.0068 (17)
C9	0.048 (2)	0.059 (2)	0.051 (2)	−0.0221 (17)	−0.0024 (18)	0.0051 (17)
C10	0.052 (2)	0.049 (2)	0.051 (2)	−0.0199 (16)	−0.0092 (18)	0.0019 (15)
C11	0.043 (2)	0.060 (2)	0.065 (2)	−0.0157 (17)	−0.0119 (19)	−0.0025 (18)
C12	0.048 (2)	0.064 (2)	0.054 (2)	−0.0247 (17)	0.0008 (18)	−0.0048 (18)

### Geometric parameters (Å, º)

**Table:**

Br1—C10	1.886 (3)	C3—H3	0.9300
O1—C5	1.204 (3)	C4—H4	0.9300
N1—C2	1.329 (4)	C6—C7	1.447 (4)
N1—C1	1.332 (3)	C6—H6	0.9300
N2—C3	1.322 (4)	C7—C8	1.383 (4)
N2—C4	1.332 (4)	C7—C12	1.393 (4)
N3—C5	1.346 (4)	C8—C9	1.370 (4)
N3—N4	1.379 (3)	C8—H8	0.9300
N3—H3A	0.8600	C9—C10	1.376 (4)
N4—C6	1.268 (4)	C9—H9	0.9300
C1—C4	1.370 (4)	C10—C11	1.377 (4)
C1—C5	1.506 (4)	C11—C12	1.382 (4)
C2—C3	1.368 (4)	C11—H11	0.9300
C2—H2	0.9300	C12—H12	0.9300
			
C2—N1—C1	115.4 (3)	N4—C6—C7	122.5 (3)
C3—N2—C4	114.6 (3)	N4—C6—H6	118.7
C5—N3—N4	121.3 (3)	C7—C6—H6	118.7
C5—N3—H3A	119.4	C8—C7—C12	117.9 (3)
N4—N3—H3A	119.4	C8—C7—C6	123.3 (3)
C6—N4—N3	114.5 (3)	C12—C7—C6	118.7 (3)
N1—C1—C4	121.5 (3)	C9—C8—C7	121.6 (3)
N1—C1—C5	118.5 (3)	C9—C8—H8	119.2
C4—C1—C5	119.9 (3)	C7—C8—H8	119.2
N1—C2—C3	122.4 (3)	C8—C9—C10	119.7 (3)
N1—C2—H2	118.8	C8—C9—H9	120.2
C3—C2—H2	118.8	C10—C9—H9	120.2
N2—C3—C2	122.8 (3)	C9—C10—C11	120.4 (3)
N2—C3—H3	118.6	C9—C10—Br1	120.6 (3)
C2—C3—H3	118.6	C11—C10—Br1	119.0 (3)
N2—C4—C1	123.2 (3)	C10—C11—C12	119.5 (3)
N2—C4—H4	118.4	C10—C11—H11	120.2
C1—C4—H4	118.4	C12—C11—H11	120.2
O1—C5—N3	125.4 (3)	C11—C12—C7	120.9 (3)
O1—C5—C1	121.7 (3)	C11—C12—H12	119.6
N3—C5—C1	112.9 (3)	C7—C12—H12	119.6
			
C5—N3—N4—C6	174.4 (3)	C4—C1—C5—N3	−173.2 (3)
C2—N1—C1—C4	−1.1 (4)	N3—N4—C6—C7	178.3 (3)
C2—N1—C1—C5	178.2 (3)	N4—C6—C7—C8	−7.6 (5)
C1—N1—C2—C3	0.3 (5)	N4—C6—C7—C12	172.5 (3)
C4—N2—C3—C2	−0.7 (5)	C12—C7—C8—C9	−0.1 (5)
N1—C2—C3—N2	0.7 (5)	C6—C7—C8—C9	−180.0 (3)
C3—N2—C4—C1	−0.1 (5)	C7—C8—C9—C10	−0.1 (5)
N1—C1—C4—N2	1.0 (5)	C8—C9—C10—C11	0.0 (5)
C5—C1—C4—N2	−178.2 (3)	C8—C9—C10—Br1	178.7 (2)
N4—N3—C5—O1	−1.9 (5)	C9—C10—C11—C12	0.4 (5)
N4—N3—C5—C1	179.1 (2)	Br1—C10—C11—C12	−178.3 (2)
N1—C1—C5—O1	−171.5 (3)	C10—C11—C12—C7	−0.6 (5)
C4—C1—C5—O1	7.8 (5)	C8—C7—C12—C11	0.5 (5)
N1—C1—C5—N3	7.6 (4)	C6—C7—C12—C11	−179.7 (3)

### Hydrogen-bond geometry (Å, º)

**Table:**

*D*—H···*A*	*D*—H	H···*A*	*D*···*A*	*D*—H···*A*
C6—H6···O1^i^	0.93	2.24	3.132 (4)	161
N3—H3*A*···N1	0.86	2.27	2.671 (3)	108

Symmetry code: (i) *x*+1, *y*, *z*.
